# Strategies to support engagement and continuity of activity during mealtimes for families living with dementia; a qualitative study

**DOI:** 10.1186/s12877-015-0120-2

**Published:** 2015-10-09

**Authors:** Heather H. Keller, Lori Schindel Martin, Sherry Dupuis, Holly Reimer, Rebecca Genoe

**Affiliations:** Department of Kinesiology, University of Waterloo, N2V 2 M9 Waterloo, Canada; Daphne Cockwell School of Nursing, Ryerson University, Toronto, Canada; Department of Recreation and Leisure Studies, University of Waterloo, Waterloo, Canada; Department of Family Relations and Applied Nutrition, University of Guelph, Guelph, Canada; Faculty of Kinesiology and Health Studies, University of Regina, Regina, Canada

**Keywords:** Dementia, Mealtimes, Family, Care partners, Qualitative

## Abstract

**Background:**

Mealtimes are an essential part of living and quality of life for everyone, including persons living with dementia. A longitudinal qualitative study provided understanding of the meaning of mealtimes for persons with dementia and their family care partners. Strategies were specifically described by families to support meaningful mealtimes. The purpose of this manuscript is to describe the strategies devised and used by these families living with dementia.

**Methods:**

A longitudinal qualitative study was undertaken to explore the meaning and experience of mealtimes for families living with dementia over a three-year period. 27 families [older person with dementia and at least one family care partner] were originally recruited from the community of South-Western Ontario. Individual and dyad interviews were conducted each year. Digitally recorded transcripts were analyzed using grounded theory methodology. Strategies were identified and categorized.

**Results:**

Strategies to support quality mealtimes were devised by families as they adapted to their evolving lives. General strategies such as living in the moment, as well as strategies specific to maintaining social engagement and continuity of mealtime activities were reported.

**Conclusions:**

In addition to nutritional benefit, family mealtimes provide important opportunities for persons with dementia and their family care partners to socially engage and continue meaningful roles. Strategies identified by participants provide a basis for further education and support to families living with dementia.

## Background

Older adults living with dementia are increasing in number and proportion with the aging population. It is estimated that there will be 65 million people with dementia worldwide by 2030, a figure expected to rise to approximately 115 million by 2050 [1]. The prediction of such a global demographic expansion results in the increased likelihood that families will support persons with dementia in the community for a longer period of time. It is therefore important that care be increasingly informed by theoretically driven empirical knowledge that translates into practice [2]. While research-informed perspectives of how to provide quality care, such as person-centred [3, 4] and relationship-centred approaches [5, 6] have been available for some time, limited attention has been paid to the importance of mealtimes for families living with dementia. Practitioners who support families living with dementia through programming and education need to base these activities on knowledge that emerges directly from research data of personal experiences.

### Nutrition and dementia

Eating and other aspects of life can change with dementia and have been shown to impact nutritional status, health, and quality of life [7, 8]. Family care partners report challenges preparing, providing, and having the person with dementia consume prepared food [9]. Most of the empirical work on nutrition and dementia has focused on identifying and preventing weight loss [10–15]. For example, alterations in capacity with cooking and shopping, food preferences, swallowing ability, and eating conduct [e.g., wandering from the table, difficulty using utensils] have been documented and associated with weight loss and malnutrition [8]. Manthorpe and Watson [16] suggest that research needs to move beyond the biomedical view of food provision, examining social relationships and interactions and how they influence food intake and quality of life for persons living with dementia.

### Mealtimes beyond nutrition

Mealtimes within the sociological perspective are defined as eating occasions with two or more people [17]. Eating with others is known to increase the amount and variety of food intake in older adults [18]. One potential mechanism is that of social facilitation which occurs when eating with others; more diverse foods are available, a longer amount of time is spent eating, and being with others can potentially improve mood, which in turn promotes increased food intake [18–21]. Thus, eating with others has a nutritional benefit.

In addition to improved food intake, shared meals are also recognized as a way in which families produce a ‘cohesive social unit’ [22]. Mealtimes provide opportunities for interaction, discussion, handling conflicts, telling and retelling family stories, and establishing and maintaining routines and identities [22–25]. For some families, meals together are a positive experience, providing opportunities to share and de-stress [25], while for others they are a source of tension due to power struggles [26]. Much of the early mealtime research has concentrated on the young family. Thus, understanding the relevance of the experience of eating with others, especially when health changes influence the way food is consumed, such as in the dementia context, is needed.

### Mealtimes in the dementia context

Community dwelling older people often experience isolation and social withdrawal in early stages of dementia [27], such that the potential benefits of eating with others may be lost. Furthermore, changes in eating conduct begin to occur while living in the community [8, 9, 28, 29], potentially leading to the person with dementia eating meals in solitude because they or others are embarrassed or worried about eating at the family table or in public. Alternatively, some couples living with dementia describe engaging in shared activities, such as meal preparation, as a way of sustaining positive aspects of their relationship [30]. It is also known that identity as expressed through food choices and food provision roles continue to be important in later life [31], evoking feelings of loss and guilt when mealtime tasks are relinquished to others [32].

Despite recognition that nutritional status and body weight are important to the progression and outcomes associated with dementia, little research focused on mealtimes has been conducted, especially in the community context [9, 30–32]. Work to date suggests challenges in coping with the many changes experienced in mealtime activities leads to caregiver stress [9, 28, 29] and this stress predicts weight loss in the person with dementia [33]. Education delivered in family caregiver support programs tends to be focused on how to change diet, maintain body weight, and access external resources [14, 34, 35]. Based on the knowledge that mealtimes are more than ‘food on the plate’, an examination of strategies to support the mealtime process developed and used by families living with dementia is needed to provide a more comprehensive basis for these education programs.

The objective of the Eating Together study [36–38] was to uncover the meaning of the mealtime experiences of persons living with dementia and their primary family care partners. Longitudinal data collection allowed us to understand what was truly meaningful for dyads as dementia progressed and transitions to formal care settings occurred. Prior publications [36–38] are focused on describing these meaningful concepts that compose the Life Nourishment Theory. Specific strategies described by the participants to enrich mealtime experiences also emerged from the data, but have yet to be reported in detail. The purpose of this paper is to present the thematic analysis of these strategies and provide in-depth description to support their use in educational programs and other activities for families living with dementia.

## Methods

### Overview of the eating together study

The Eating Together Study was a longitudinal qualitative investigation, using grounded theory methodology [39] to develop a substantive theory on the meaning making process of mealtimes for persons with dementia and their closest family partners in care [36–38]. Potential participants were recruited from Alzheimer Society Chapters in South-Western Ontario; chapter staff was provided information on the study and notification was advertised in chapter newsletters and communications. Purposive and theoretical recruitment occurred to promote inclusion of male and child care partners, whereby chapter staff provided study information directly to these groups. Family members who were interested in the study, contacted the researchers for more information and were invited to participate if they were eligible. Eligible participants were those where: both members of the dyad were willing to be interviewed; spoke English; persons with dementia were 55 years of age or older and in the early to mid-stages of dementia; and the primary family care partner and person with dementia lived in the community.

Twenty-six dyads and one triad were interviewed individually and as a dyad on an annual basis for three years. The initial sample included 11 male [41 %] and 16 female [59 %] persons living with dementia ranging in age from 56–88 years. Care partners varied in age between 30 and 88 years of age. Twelve [43 %] were male and 16 [57 %] were female. There were 19 spousal relationships and eight adult-child relationships, including three daughters, three sons, one niece, and one daughter-in-law; 24 lived with the person with dementia. All families were Caucasian and predominately of European descent. There were no families represented that had specific religious beliefs that impacted food behaviours. The Functional Assessment Scale [40] was used to determine stage of dementia and re-evaluated each year. Over the three years, persons with dementia progressed; for example, in Year 1, 20 had a Functional Assessment Scale [FAST] rating between 1 and 4, whereas only 10 remained at this level by Year 3 [see Table 1]. Type of dementia or specific diagnosis was not ascertained. By the end of three years, 18 dyads were still available for interviews and where possible, interviews continued with both partners after long term care placement.

**Table 1:** Characteristics of eating together participants

Characteristic	Year 1 [*n* = 27 families]	Year 2 [*n* = 23 families]	Year 3 [*n* = 18 families]
*Gender*			
PWD [*n* = 27]	11 M: 16 F	10 M: 13 F	7 M: 11 F
CP [*n* = 28]	12 M: 16 F	10 M: 13 F	8 M: 10 F
Caring Relationship			
Spouse	19	19	14
Child	6	3	3
Niece	1	1	1
Daughter-in-Law	1	0	0
FAST			
1–4	20	15	10
5-6C	5	5	5
6D-7 F	2	3	3
PWD Age range [yrs]	56-88	---	---
CP Age range [yrs]	30-88	---	---
Residence			
Community	27	17	13
[Live with PWD]	[24]	[16]	[12]
Facility with spouse	0	3	1
Facility PWD alone	0	3	4
Reason for Leaving Study	N/A	[4 lost in Yr 2]	[5 lost in Yr 3]
Death of PWD		2	
Death of Spouse		0	2
Moved/Lost Contact		2	2
Refusal		0	1

*PWD* person with dementia, *CP* family care partner, *FAST* functional assessment scale *M* male, *F* female, *N/A* not applicable

Five trained interviewers conducted dyadic active interviews [41], followed by individual interviews with both the person with dementia and the care partner approximately two to four weeks later. Questions were focused on how mealtimes occurred for the family, and the meaning of these experiences. As the theory unfolded, questioning focused on filling out conceptual categories (e.g. how do you promote connecting at mealtimes). Specific to the purpose of this paper, when participants identified a problem that had occurred with respect to mealtimes, questioning was used to identify any strategies the person with dementia or their care partner employed to address the challenge. Individual interviews provided an opportunity for each member of the dyad to further elaborate on their perspective, which may not have been fully flushed out during the dyad interview. It also allowed the interviewer and participant to follow up on potentially sensitive topics raised in the dyad interview. Active interviews [41] were conversational in style and involved mutual disclosure. All interviews were digitally recorded, transcribed and checked for accuracy by trained transcriptionists and research associates. Approximately one and two years later, the dyads were interviewed again, following the same procedures. Verbal and/or written consent were provided by both persons with dementia and partners in care as approved through review by the Research Ethics Boards of three academic institutions (University of Guelph, Univeristy of Waterloo and Ryerson University). Anonymity was ensured by use of dyad numbers to code and represent data. All data were securely stored to also ensure confidentiality.

Following transcription, interviews were analyzed by individual interviewers and the team, concurrent with on-going data collection. Consistent with grounded theory methodology, constant comparison and coding techniques [initial, focused, axial] were used [39]. Nvivo was used to track the coding structure and support analysis. Team meetings were regularly conducted to develop the line of questioning. Memos, conceptual maps and key theme documents were used to relay individual analysis to the other team members during these meetings and support further analysis as a group. This helped to reduce bias of individual researchers on the interpretation of the data. The longitudinal analysis allowed us to not only refine themes due to evolving lines of questioning, but also to reflect and understand the experience of the changing mealtimes for families as dementia progressed. Strategies derived were validated by an independent group of persons with dementia who were involved in creating a *By Us For Us* guide on mealtimes and food [42]. Further details on methodology have been published in prior manuscripts that describe in depth the three underlying concepts of the theory [36–38].

## Results

### Brief overview of the Life Nourishment Theory

The study findings resulted in the emergence of a substantive theory built upon three inter-related concepts, *being connected, honouring identity,* and *adapting to an evolving life,* that made mealtimes meaningful to families. Each of these concepts is the focus of prior publications [36–38]. Mealtimes provided a natural, normative and guaranteed time for dyad participants to connect with each other and friends and family. Mealtimes were seen as important social events, potentially more so because of dementia [36]. Our research also identified that mealtime routines and traditions helped participants to honour individual and family identities [37]. As abilities changed, honouring identity involved a delicate balancing act between control and capacity. This was important for nurturing the personhood of the person with dementia [PWD], as well as the care partner [CP]. Families were faced with many changes that significantly impacted the way their lives were lived. Changes were often subtle, but incessant and required constant adaptation [38].

### Strategies to support the meaning of mealtimes

Interviewing was focused on uncovering and deepening the understanding of the concepts in the Life Nourishment Theory (LNT), thus challenges and changes families were experiencing were focused on the concepts of staying connected, honouring identity and adapting to their evolving life. This resulted in a line of inquiry to understand how the dyad moved through the change, eliciting examples of a variety of strategies used to maintain meaning at mealtimes. General coping strategies were described and included: adopting a positive attitude, having a sense of humour, reframing the problem, gathering information/accessing external resources, focusing on strengths, developing and sustaining trust, doing things together, minimizing risks, being realistic, simplifying tasks and living in the moment. Strategies specific to some of the concepts of the LNT are presented in Table 2. These strategies can be summarized as those that supported *mealtime social engagement* and those that supported *continuity of mealtime activities.*

**Table 2:** Data driven strategies to support mealtime social engagement and continuity for families living with dementia

Key LNT concepts	Strategies
Taking time, Focusing attention	• Make meals an important ritual in the day, not a task; avoid competing activities and interruptions
	• Sit and eat together
	• Provide sufficient time to eat in a calm environment
	• Eat out of the home sometimes, away from distractions of meal preparation
	• Focus on making the dining experience calm and relaxed
Communicating activities, staying informed, gain knowledge, share and create stories	• Use conversation aids e.g. the environment, the food, letters and messages from family and friends
	• Talk about the day
	• Reminisce
	• Support communication by prompting around names, summarizing conversation etc.
Making decisions	• Provide options when grocery shopping, making meals and eating out
	• Discuss issues/plans
Emotional support	• Be appreciative and encouraging
	• Give full attention, listen
	• Be easy-going; use humor
	• Check in with genuine care
	• Go on special eating outings to alleviate daily stress
	• Share burdens
Physical support	• Provide assistance as needed with meal preparation and eating
	• Simplify the menu, select meals that are easy to make and eat
	• Take-out/pot-luck for entertaining
	• Access external resources to provide support when needed
Psychological support	• Talk about the food and things you can see
	• Ask questions that are focused on opinions or preferences
	• Gently redirect if conversation is repetitive or help to identify words as needed
	• Recognize that listening is also participation
	• Rehearse names and connections before getting together with others
	• Sit near a window, listen to the radio, read letters/emails together to provide topics for conversation
	• Help make decisions about menu choices when eating out
Taking part, enabling and negotiating roles	• Recognize the meaningfulness of individual mealtime tasks including feeding oneself
	• Share mealtime tasks or supervise and let others take on roles
	• Be flexible; recognize daily differences in capacity and interest
	• Breakdown tasks and match abilities to tasks
	• Provide opportunities for repetitive activities that are meaningful
	• Discuss, observe ways that a role can be adapted but still accomplished
Being creative	• Make meals attractive
	• Spend time planning and discussing meals together
	• Food is a common interest that is retained throughout life; use it to stimulate interest
	• Try new foods and recipes
Being accepted, acknowledged, veiling reality	• Understand that change is inevitable; flex and transform
	• Focus on supporting connection and dignity
	• Focus on current strengths and overlook mistakes or missteps
	• See the individual, not the disease or the activity
	• Provide praise and encouragement; be appreciative for contributions
	• Seek to understand opinions and desires
	• Leave things that are difficult or challenging ‘unsaid’; protect dignity
	• Avoid making others feel self-conscious or embarrassed [e.g. if appetite is poor reduce portion size]
	• Show respect for choices
	• Be aware of and meet preferences
Promote routines and traditions	• Keep meal routines and traditions as much as possible [e.g. where to sit, timing, and process of the meal]
	• Identify essential aspects of traditions that need to be retained as changes happen; adapt less essential components
	• Replace less meaningful tasks with new routines and traditions that support engagement and continuity of identity

### Mealtime social engagement

Mealtimes provided a time when social interaction was often easier for families. Specifically, the activity of eating provided the opportunity to be engaged in socializing without need for constant flow of conversation. By virtue of the act of eating, persons with dementia could contribute to the relational component of mealtimes by listening rather than feeling obligated to be verbally active. As well, the physical space and closeness afforded around a dining table provided the time and proximity for conversation to happen, more so than at other points in the day. Thus, strategies to remain engaged for both members of the dyad were commonly discussed in interviews.

Conversation could be somewhat stagnant at mealtimes due to memory challenges, and for spousal dyads, spending much of the day together. Conversation aids were commonly used to stimulate interest and introduce new topics. Families described supporting mealtime conversation by sharing information and asking for opinions from the person with dementia. Reading letters or emails from family was one specific strategy used by Dyad 6, to promote connections with their extended family. This family also used the technique of a fishbowl filled with short messages from family members which sat on the dining table; these were used to share memories or feelings of the family with the person with dementia.

CP6: … it could be our children or their children, to write down different memories they had of times with us. And so they, all these different colours represent different families. And so they were all printed off and cut in strips and folded and in this [bowl], and we were given that as a Christmas gift. PWD6: And a full bowl. CP6: Oh yes, it was way up to there [gestures]…[PWD’s name] says, ‘Well let’s leave it on the table and then we’ll remember to do it.’

At meals the care partner would prompt her husband to select and read out a message at the table as a way to start a mealtime conversation.

Other conversation aids could include the physical environment or the food itself. Birds outside the window [dyad 10] at a feeder or discussion of a new recipe helped to stimulate dialogue for the dyad [dyad 3]. Sometimes, the television or radio was used to provide the sense of ‘company’ at the meal or stimulate interest and conversation. These conversation aids provided opportunities for viewpoints or opinions to be expressed that did not rely on memory. Reminiscing was another common strategy that not only supported interaction, but also identity. Sometimes specific foods were used to trigger reminiscence and spark a conversation [e.g., the taste of citron was a memory that lead to conversation around childhood foods for dyad 16].

Providing ample opportunities to make decisions throughout the mealtime activity [e.g. choosing tasks to partake in, what foods to consume] also promoted conversation and engagement, but also protected the dignity of the person living with dementia. Family or friends could support the making of these decisions:

… the restaurants we go to – [my friends and family] pretty well know [what I like to eat]. And…whoever is sitting beside me knows that I want something [with] fish…Remembering the last time I was out – I don’t remember who was beside me but probably [they] did say, “What fish do you want?” PWD, dyad 9

Care partners also supported the person with dementia staying engaged at the meal by slowing down the pace of conversation, helping to find words, taking time to listen, saying names of members at the table when conversing, and recapping of a storyline told by others so that the person with dementia could follow along. Using these strategies discretely protected their dignity. For example,

…obviously her collection of friends are very much aware and they will bring PWD9 in [to conversation] because they would say, “[PWD’s name]”; they would identify the events and they would bring her in [to the conversation] as what she did or didn’t do or whatever. There may or may not be a recollection [by the person with dementia] but they’re very perceptive that way. CP, dyad 9.

In this instance the support afforded to the person with dementia, extended beyond the dyad and demonstrated that such assistance helped this woman to remain socially engaged with her strong network of friends.

Families living with dementia enjoyed socializing by eating out, or getting together with family or other social groups, as a way to de-stress and stay connected. However, as dementia progressed this could be more challenging: “It’s just, it’s too difficult because I have to…wonder what PWD25’s up to. If she’s.....she’s restless when there are a lot of people around ” [CP25].

Social engagement at mealtimes was often facilitated by a calmer atmosphere. Limiting exposure time or number of people or taking a break and seeking a quiet space to leave the conversation for a while, were strategies dyads used to remain socially engaged with others. As dementia progressed, reducing the size of the groups or gatherings for meals together was a common strategy to reduce stress for both members of the dyad. Disclosure of the challenges being experienced to a supportive social circle also helped care partners and persons with dementia to feel comfortable.

Some families described rehearsing names and associations before visiting with friends and family so that the person with dementia would be able to participate more fully, or provided reminders on etiquette. For example: “Well, in social situations I…like before we go somewhere I have to maybe remind her …about slow down when you’re eating” [CP 18]. However, to protect dignity as well as to decrease risk of confrontations at group events, sometimes care partners intervened, tried to help others to understand the needs of the person with dementia, or encouraged others to overlook the challenges the person with dementia may have been having. In the case of dyad 18, the wife who had frontal-temporal dementia, was quite outspoken with family and would sometimes need her husband to counter her comments: “Well, if the whole group is talking about a particular subject and she’s giving information that’s not correct, on the topic then I sometimes go…try to excuse it by saying ‘I think that might not be right.’ Try to do it diplomatically” [CP18].

Entertaining or hosting others for a meal also changed over time for all dyads. A couple who lived in a retirement community were involved in many of the social activities of the housing complex. However, over time hosting these events had to be handed over to others, due to the demands of caregiving.

It’s a bit of a pain. We used to always have at least a couple of garden parties in the summer time and I barbecue a whole bunch of stuff for a lot people. Tennis bunch and what not. We don’t host it anymore…The whole thing is kinda collapsing around us, our social life. CP25

Although not always a preferred strategy, reducing or eliminating entertaining was commonly discussed. Yet, family events were often prioritized. In spousal dyads where they had previously done much of the hosting for the extended family, this changed with the progression of dementia. Extended family picked up the majority of the tasks for traditional celebrations so that most of the work of planning and completing entertaining tasks was not on the spousal couple experiencing dementia. For example, a care partner in a spousal dyad that was used to hosting large family gatherings noted:

No, we went …to our oldest daughter’s. And so she got to do the planning and the orchestrating and did a wonderful job and we weren’t all that involved in it this year. Of course that was the idea when they started the planning. They said, ‘Ok, we want you to do this but not necessarily where you get to do all the work’. CP6

Alternatively, the couple could take family members and friends out to restaurants as a way of fulfilling their desired role of ‘hosting’ others for a meal. In a family [dyad 9] where the wife who was an excellent and creative cook who had early onset dementia, they used this strategy as her capacity for cooking waned. When asked what she thought of this adaptation she stated:

Well, you’re probably wondering does it bother me? It doesn’t. No. Again no. Don’t know why but I’ve just sort of accepted this and that’s the way it is. It doesn’t bother me. It’s the fact that I can still, you know they still wanna come and they come. So what the heck, do they care what their eating? No. And I’m feeding my man, I’m feeding the kids. They’re happy; they don’t care if Nana made it or if…they’re probably just as happy it came from McDonalds. I know that. So I know that but it’s the fact that we sit around together. PWD9

This quote exemplifies how many families felt about the meaning of mealtimes, and how they adjusted and strategized to make the most of mealtimes as a social experience where they could remain engaged with loved ones.

### Continuity of mealtime activities

Mealtime activities include grocery shopping [including driving, selecting foods, pushing the cart, paying for the food, loading the car, getting home, carrying groceries, and putting groceries away], preparing food [including deciding what to make, following a recipe if needed, washing, stirring, measuring, chopping, combining, using the oven or fridge], setting the table [determining number of persons eating, what utensils/dishes needed, placement], consuming the food [cutting if required, choosing utensils, moving food to mouth, eating without spilling], and cleanup [clearing dishes, washing or putting in dishwasher, drying, putting dishes away] and the multiple smaller steps that each task encompasses. Completion of these activities provided a sense of accomplishment for both care partners and persons with dementia and also a point for connection, if done together.

But she [PWD 23] loves to be engaged in something like that. So if there is a time when I need to do something, and I can’t kind of supervise two things at once. Like she’s quite anxious to, “Give me a job to do.” you know. So it’s, I’m, I do try to think ahead about things that I can - give her and keep her involved in. Yeah. CP23

In addition to feeling like one was contributing and being involved in meaningful tasks for the household or family, participating in meal activities were often tied to the continuity of personal identity.

Wives and/or mothers with dementia commonly saw mealtime activities as part of their role. Strategies were used by families to help negotiate changes to that role while retaining dignity. Including the person with dementia as part of the decision making process was a key strategy to support personhood. For example, this wife with dementia recognized her changing abilities as well as her husband’s capacity:

I was, we talked about it and he thought it will be a good idea [to learn to cook]. Because we both know that my mind is, I have Alzheimer’s and I thought that was a good idea that he knew how to cook. And actually he enjoys cooking so it works out quite well. PWD19

Such realizations were not always automatic or smooth and negotiating capacity to complete activities was often discussed in interviews. This negotiation was habitually in the moment based on current needs, feelings and where both persons in the dyad were currently ‘at’. For example, this mother with dementia who lived with her married son liked to be involved in meals and cooking traditional family dishes, but often she was not up to the task of focusing or standing over the heat of a stove, so negotiating day-to-day was a strategy they used to promote continuity in mealtime tasks:

Just you know, it wasn’t pre-planned or anything – [it] just happened. CP5 comes home [from the store] and she had all this meat and I said, “Well, I can help.” And we just did it and I’m glad that it was a good day for me…And I know a lot of people under that – in that scenario when they’ve had all that to do – really, [it] would have been quicker feasibly to do it on their own. But when we started I didn’t actually slow the process. It just happened to work really good that day because it was a good day for me. PWD5

Capacity also needed to be negotiated for care partners who had their own health issues. For example, care partner 16 commonly made homemade desserts for their extended, blended family on weekends and when family came for dinner. However as her spouse’s dementia progressed and her own health changed with a cancer diagnosis, her capacity for these activities decreased and she needed to identify what was most important about traditions that needed to be retained, while letting go of other aspects.

We decided to go potluck. Which was really nice. I found such a release from that. Until I reminded myself that I usually make an Easter Bunny cake tradition. And I thought. ‘Oooooh, thank goodness it’s just a fast cake mix’ because I thought, that’s the trouble with traditions. Can force yourself into stress. But what, what freed me was my daughter-in-law made the turkey. CP16

Thus, negotiation of capacity is dynamic and includes capacity of both members of the dyad as well as others who might be sharing the meal.

Care partners worked to support the person with dementia to retain mealtime activities. For example, they supervised in the kitchen or helped with planning, recipe reading, measuring, and keeping track of cooking time. As described by this wife with dementia, this support was essential to helping her achieve her goals of social engagement:

I don’t know how people that have Alzheimer’s do it on their own because it’s very difficult. My back-up is CP18 and you need that too. Like I wouldn’t be able to entertain the way I do if it wasn’t for him. PWD 18

As dementia progressed, specific tasks and activities were managed within the dyad so that dignity was upheld but also capacity was not exceeded. For example, the female spouse in dyad 9 had dementia and was a creative cook with a strong identity linked to food preparation. In the early stages of dementia, she continued to plan meals and host family events. However it was evident that some aspects of this role were a problem for her:

I think we’ve talked before about the difficulty of having a large group …now she needs to be more or less looking after certain elements of the meal and she’s quite efficient at that. But getting the meal on time and getting people to sit down on time that’s probably a little more anxious [for her] than maybe in the past. CP9

As her dementia progressed, family gradually managed her changing capacity with respect to entertaining and chose to go to a club or restaurant for meals with her husband’s work associates. For family gatherings, their daughter who lived in the same neighbourhood, would come over to her mother’s and prepare the main dishes, leaving the final creative touches to her mother for table settings and vegetable dishes. As noted by this mother with dementia when asked what she thought about this change in role:

So no, it’s not a problem. And I think because they all know how I feel about my vegetables that I don’t think anyone would ever – well no one likes to do vegetables anyway. So if I like doing them that’s cool. PWD9

Although sometimes frustrations were noted by family members, care partners described that using diplomacy was a more successful strategy when negotiating changing capacity.

Another strategy was considering and using external resources when needed to achieve one’s goals. This could be family helping with traditional family-get-togethers or community services. As stated by this person with dementia who wanted to stay at home alone while her care partners were on vacation:

I was kinda looking forward actually to be in the house myself although there’s certain areas I don’t function very well and obviously that’s the meals. So we did that, we did make alternate arrangements [getting home delivered meals] for that period when it came to the meals. PWD5

A variety of external resources could be used especially for cooking and shopping, including taxis, tabs at grocery stores and community service homemakers who could support food preparation by the person with dementia. Purchasing semi prepared foods to reduce effort and time was also an example of using external resources.

Sharing tasks was a key strategy for success. For example, with grocery shopping, care partners helped with making lists, provided opportunities to be engaged in food selections, but when necessary did not depend on the person with dementia for all of these decisions. Care partners understood the importance of sharing in the work and keeping the person with dementia involved, and letting them decide how much and when they wanted to be involved.

CP18: I don’t say, ‘Hey, stop that I’ll do it.’ Like I still let her do all of the work. It’s important that she does all the work; I just do all the reading of the recipe and make sure that order is kept. Like she has the…if I was to let her alone, she reads the recipe, or she reads the ingredients and she doesn’t add them in the order that they’re listed and things and she doesn’t realize that this has a real consequence on the final product. [Laughing]. PWD18: Timing of the cooking he does that. He makes sure that the stoves are off; the oven is off everything after that. And he makes sure… he looks after the meat usually. And then….it’s just to keep everything proper.

Every mealtime activity had an opportunity for most persons with dementia to be involved if they wanted, as there are several discrete tasks that are part of the activity, such as pushing the cart in the grocery store. Understanding that these activities had many tasks or steps and matching these to current capacity was a way of promoting the continuation of mealtime activities.

Reminders, detailed guidance and routines were used by care partners to retain involvement of the person with dementia. For example, a mother and daughter [dyad 23] that lived separately, shopped together once a week. It was important to the mother to remain independent for as long as possible, including paying for her own groceries. Sometimes when she forgot the personal identification number for her bank card, her daughter would give her a clue that was the basis for this code: “Yes and I, and I usually have to remind you. Or you check it out with me. [PWD23 agrees] Cause you know what the code is” [CP23]. Providing more detailed instructions or guidance as well as breaking down tasks into individual steps were ways of helping the person with dementia to stay involved. For example, a wife who still worked, would prepare her husband’s lunch, but would leave him instructions to support his ability to reheat the meal:

PWD6: A note on the table there says ‘Put it in the microwave for that many minutes’ or so forth. And so that’s what I go by anyway whether you are here or not. …CG6: And the other thing is I don’t really know whether he will stop and think about ‘Now, I heated up something like this last week. How much did I heat it for then?’ So that sort of thing isn’t, that thought process isn’t there. And so I just try to make life easy for him. Take out anything that might be complicated.

Detailed instructions were also used when making grocery shopping lists. Some persons with dementia tended to over purchase the same products, especially if they were on sale. Making a list of what was already in the cupboard, was a way of controlling these purchases.

Repetitive tasks and routines also helped persons with dementia to not become overwhelmed:

And what I was doing was not, not that demanding [tenderizing meat]. So it wasn’t a bunch of things, it was like one thing repetitive. So that made it easy for me to do rather than having my brain trying to think of every step all along the way…because it was, every thing was so repetitive it went really, really well for me. So it turned out to be a good day and we got a lot accomplished and I must admit I felt I held my own which doesn’t usually happen in the kitchen. PWD5

Routines such as the time meals were served or order of activities helped to support continuity of mealtime activities. For example, to support grocery shopping, CP9 arranged for his wife with dementia to have a tab at a local store so that she did not need to carry money. As part of her routine, she walked to the store most days of the week to select items for the evening meal.

A further strategy to continue mealtime activities for the person with dementia was to capitalize on current strengths such as physical ability. Some wives found that their husbands with dementia, who had never participated in making meals before, hung out in the kitchen to be close to them. They put these men to work doing the heavier tasks such as chopping or lifting things out of the oven.

CG16: No. He’s been very involved. If I’m preparing anything. Like I made a lot of pies at Easter time. And stuff like that. He’s very – I’ve, I’ve needed his assistance more – chopping things if I’m making anything. Opening. I get him to open a can – like even last night. That’s what we had last night! We had a salmon sandwich for supper! [Laughs] PWD16: I don’t feel I’m being asked to do something special. I feel we’re working together at it.

The men felt useful and were being helpful to their wives when they were involved in this work. As dementia progressed, the person with dementia was often less interested in shopping and cooking and would shift to the role of supervisor or observer for mealtime activities. For example, a woman with dementia who was a good cook and involved in their social group with entertaining large groups of people, found a new role as interpreter and supervisor, capitalizing on her current strength:

…they [church group] ask her to give them a hand in the kitchen to prepare whatever they are doing. Now she’s not doing very well [with cooking] like she was doing on the beginning. … And they didn’t speak English so PWD20 she was doing the interpreter in English and Italian. CP 20

Activity specific strategies were also commonly developed to remove or reduce the stress of mealtime activities, whether it was grocery shopping, eating out or entertaining. For example, grocery shopping was done at quieter times. Older appliances were retained as they were simpler to operate. Taking family out for a meal was a way for the person with dementia to feel like they were still hosting: “Because - it’s much easier than preparing a meal and entertaining a guest, and being part of the proceedings whereas if the meal is looked after then I can enjoy it a lot, a lot more” [PWD23]. As noisy or busy restaurants could be overwhelming, choosing those that had a calm atmosphere or going at times when they were less busy, helped families negotiate this environment. When entertaining was still done at home, menus were simplified, take-out or semi-prepared foods were used, and some moved to sharing the workload with guests either by the assembling dishes together or having them bring a dish.

Sometimes, the experience of a stressful situation for the care partner prompted different arrangements being made so that dignity of the person with dementia could be upheld. A care partner who was dealing with her own health issues agreed to homemaking to support meals and facilitate keeping her husband involved in activities:

Well, actually that’s where I have to be very careful. Is ah, he can help me, but helping him. [Laughs] I know my frustration level. [Laughs] Ah, I have to kinda bow out in, in and believe that he can do what he can do. And it’s very helpful. This is actually why he has a [personal support worker]…..And I know to stay out of it. And that, that I guess, the dignity part. Knowing that he can do it. [Right] And stay out of it…… Because I might get - that’s where I know my patience and I know my weakness area, that that’s where I have to be very careful. CP16

Some care partners and extended family would specifically leave a task for the person with dementia to do, especially when they knew the person with dementia would ask to be involved. For example:

Yah, and [family member] will have things in pretty good shape but she won’t have all the dishes done or whatever. The dishes she’d have them done but they’re just sitting to be put away or dried or something. And so PWD10 she gets right at it and she cleans this dishes and she puts them away. Of course [family member] has to go afterwards and change them around. She doesn’t mind at all but she thinks it’s really helpful for PWD10 to do that. CG10

When tasks were initiated by the person with dementia but not finished or done correctly, the care partner would finish privately to preserve dignity of the person with dementia. Care partners soon learned not to jump in too soon to provide help or to fix the problem as well as to not criticize the person with dementia. This meant that more time was often needed for mealtime activities. The pace needed to be changed, such as preparing part of the meal or dish one day and finishing the next. All of these strategies were put into place to promote continuity of the mealtime activities the person with dementia was interested in being engaged in, while supporting their dignity.

## Discussion

Communal eating acts as the social setting for listening, bonding and comforting [43], and is important for group membership and marking inclusive social relationships [23]. Mealtimes have also been described as a place for role completion and development, identity formation and socialization of positive values or principles through conversation and actions [25, 26]. Furthermore, it has been identified that mealtimes might be the ‘setting where transitions [in family life] are keenly seen’ ([26], p. 86). Connectedness and sense of self provide meaning in aging [44] and thus it is relevant to consider how meals and the strategies used to continue social interactions and mealtime role engagement can be supported for families living with dementia. In this study, the perspectives of persons with dementia and their primary family partners in care were provided in yearly dyad and individual interviews for three years, resulting in an in-depth understanding of their mealtime experiences and strategies used to support mealtime social engagement and continuity of mealtime activities.

This study not only identified general coping strategies like simplifying, but also unique strategies that promoted meaningful mealtimes for family and specifically how to stay socially engaged and continue mealtime routines and activities as dementia progressed. Strategies to support social engagement included: using conversation aids; finding ways to promote decisions; reminiscing; aiding the person with dementia to be part of group conversations; finding ways to overcome social challenges; and sharing tasks for hosting others or using eating at restaurants as a way of hosting. The goal of maintaining involvement in mealtime activities, especially to promote dignity, was supported by: negotiating capacity for tasks on a day-to-day basis; providing supervision and assistance as needed; using diplomacy and being sensitive to desired roles and how to support them; sharing tasks, but keeping the person with dementia involved; using external resources including, such as semi prepared food; reminders and detailed guidance; focusing the person with dementia on repetitive tasks; and using current strengths and adapting tasks.

The global strategies identified in this study are consistent with prior work [9, 28]. However the strategies to support social engagement and mealtime activities are unique to this study. We contend that these theoretically based strategies that are consistently and purposefully implemented during mealtimes will serve to honor identity and facilitate continued connections. Strategies that spanned the varied food related activities of grocery shopping, cooking and eating. Furthermore, examples of strategies provided here are problem focused, emotion focused, and meaning-based [45], demonstrating that families use a range of strategies when coping with stressful situations.

Most intervention research to date has emphasized eating behaviours and improving food intake and nutritional status of persons with dementia [14, 34, 35, 46]. When discussing mealtime activities and behaviours, a negative discourse is often used, focusing on decline [28] and using terms such as ‘aversive eating behaviors’ [29]. These prior investigations, which focus in on a pattern of decline in food related activities, offer little insight into strategies beyond sharing responsibilities, simplifying food preparation, and using routines [9, 28]. Presenting a generally negative view of eating and dementia and using strategies that exclude the person with dementia [34, 35] is also not useful for planning educational programs for families. Although these authors identify how families moved through and began to cope with the changes they were experiencing, they also described punitive strategies used by families such as removing access to food [9], avoiding eating out of the home [28] and avoiding eating with the person with dementia [29]. The focus of devised strategies tends to be on what is eaten [for nutrition, maintain body weight] [14] and how it is eaten [29], rather than the potential other benefits of meals for both the person living with dementia and their family members. Yet, some of these authors also acknowledge the importance of the meal and what it means to family functioning and dynamics [9, 28].

The novel findings from the Eating Together study extend this prior work on strategies, providing more specific examples and greater detail that are proactive and support personhood and dignity. By focusing strategies away from the food itself and body weight, other positive outcomes of shared meals for families living with dementia can be realized, such as companionship and the nurturance of a sense of self. Focusing on the meaning of the mealtime experience and how this can be supported, also offers a different view of how families learn to adapt when a member is experiencing dementia; it provides hope and demonstrates that caring can be fulfilling [47].

Prior work also suggests that eating behaviours [e.g., eating with hands] are associated with not only poorer nutritional status of the person with dementia, but also increased caregiver *“burden”* [48, 49]. Behavioural interventions in LTC, such as involving persons with dementia in meal preparation, have been shown to support social engagement, self-feeding and may improve food intake [50]. We contend that targeting social engagement and continuity of mealtime activities with a variety of strategies taught to family care partners living in the community may be a means of improving nutritional status and mitigating stress experienced by both persons living with dementia and their care partners as well. Future intervention studies with families living in the community could be developed to include these components.

### Strengths and limitations

A unique aspect of this work is that it is longitudinal and includes a larger number of participants and more diverse dyad relationships than similar qualitative work [9, 28, 29]. To our knowledge, similar qualitative studies focused on mealtimes are rare and this is the only report to include the voices of persons with dementia. It is important to gain the perspective of the person with dementia, especially around their experiences and how they adapt to the many changes that occur with an illness causing dementia. In this work we had the opportunity to hear how families found creative ways to adapt so as to maintain valued activities, relationships, and identities, as well as the dignity of the person living with dementia. Such perspectives provide a very different picture from work to date, which has focused in on the physiological and cognitive changes, typically noting only the challenges experienced at mealtimes. Despite these strengths to this work, there are some limitations. All families were Caucasian and thus the varied experiences of other ethnic groups is missing. Families who agreed to participate and stayed in the study may have been different from the average family living with dementia; they had may have had superior coping skills and strong internal and external resources that supported resilience.

### Relevance

Care partners in prior research describe being uninformed about the nutritional needs of persons with dementia and the many and diverse challenges they are likely to experience and how these can be managed [9,51]. In response to this lack of knowledge, a variety of educational programs or interventions have been developed. Shatenstein et al., [14] provided individualized counseling to care partners specific to nutritional goals [e.g. increase fruit and vegetable intake] of persons with dementia, while others [34, 35] offered a standardized program with several sessions focused on key topics for families living with dementia, such as the importance of maintaining body weight, how to compensate for low intake, and general coping strategies. Education and self-management programs that include the person with dementia are also needed. Potential topics include: understanding of how to make mealtimes meaningful for the individual; focusing on strategies that help to realize the potential of mealtimes; promoting personhood and understanding the unique desires of the individual; nurturing relationships; and using mealtime activities as a way to enhance quality of life. These areas could be readily translated into existing programs and modeled by peer and professional care partners.

## Conclusions

It is clear from this work that older persons with dementia and their family care partners take action to sustain social engagement for both members of the dyad, as well as continuity of food and mealtime roles, routines and traditions. Diverse general strategies [such as patience and planning ahead] and more specific strategies focused on key mealtime activities and individual tasks were discussed by dyads during this three-year study. Goals of identity, continuity and social engagement were attained through adjusting to incessant change. Involving persons with dementia in family meal preparation can serve to sustain personal identity and role mastery/satisfaction far into the progression of the disease. Thus, the planning for, preparation of, and sharing of meals should involve persons with dementia across the span of this illness. Strategies that our study participants used to encourage mealtime involvement and support meaningful mealtimes should be taught to both professional care partners, peer leaders and family care partners as well as emphasizing that mealtimes provide more to families than just food on the plate.

## Abbreviations

CP: Care partner
FAST: Functional assessment scale
F: Female
M: Male
PWD: Person with dementia
